# Resorcinol–triethyl­enediamine (1/1)

**DOI:** 10.1107/S1600536811029308

**Published:** 2011-07-30

**Authors:** Yi-Hong Gao

**Affiliations:** a College of Chemistry & Chemical Engineering, Xianyang Nomal University, Xianyang 712000, People’s Republic of China

## Abstract

The title co-crystal, C_6_H_12_N_2_·C_6_H_6_O_2_, is composed of neutral resorcinol and triethyl­enediamine mol­ecules in which the resorcinol mol­ecules came from the *in situ* deca­rboxylation of 2,4-dihy­droxy­benzoic acid. In the crystal, the components are connected by O—H⋯N hydrogen bonds, forming a chain in the *b*-axis direction.

## Related literature

For background to alkali metal bis­(salicylato)borates, see: Barthel *et al.* (2000 ▶) and to organic base bis­(salicylato)borates, see: Han *et al.* (2007 ▶); Li & Liu (2006 ▶).
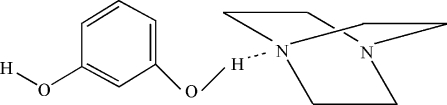


## Experimental

### 

#### Crystal data

C_6_H_12_N_2_·C_6_H_6_O_2_
*M* *_r_* = 222.29
Monoclinic, 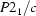

*a* = 9.4882 (5) Å
*b* = 23.7390 (11) Å
*c* = 11.2532 (6) Å
β = 113.335 (6)°
*V* = 2327.3 (2) Å^3^
*Z* = 8
Mo *K*α radiation
μ = 0.09 mm^−1^
*T* = 293 K
0.45 × 0.43 × 0.02 mm

#### Data collection

Oxford Diffraction Xcalibur Eos Gemini diffractometer
Absorption correction: multi-scan (*CrysAlis PRO*; Oxford Diffraction, 2010 ▶) *T*_min_ = 0.789, *T*_max_ = 1.000
8717 measured reflections
4091 independent reflections
3270 reflections with *I* > 2σ(*I*)
*R*_int_ = 0.017

#### Refinement

*R*[*F* ^2^ > 2σ(*F* ^2^)] = 0.045
*wR*(*F* ^2^) = 0.121
*S* = 1.03
4091 reflections
290 parameters
H-atom parameters constrained
Δρ_max_ = 0.19 e Å^−3^
Δρ_min_ = −0.16 e Å^−3^

### 

Data collection: *CrysAlis PRO* (Oxford Diffraction, 2010 ▶); cell refinement: *CrysAlis PRO*; data reduction: *CrysAlis PRO*; program(s) used to solve structure: *SHELXS97* (Sheldrick, 2008 ▶); program(s) used to refine structure: *SHELXL97* (Sheldrick, 2008 ▶); molecular graphics: *SHELXTL/PC* (Sheldrick, 2008 ▶); software used to prepare material for publication: *publCIF* (Westrip, 2010 ▶).

## Supplementary Material

Crystal structure: contains datablock(s) I, global. DOI: 10.1107/S1600536811029308/vm2111sup1.cif

Structure factors: contains datablock(s) I. DOI: 10.1107/S1600536811029308/vm2111Isup2.hkl

Additional supplementary materials:  crystallographic information; 3D view; checkCIF report

## Figures and Tables

**Table 1:** Hydrogen-bond geometry (Å, °)

*D*—H⋯*A*	*D*—H	H⋯*A*	*D*⋯*A*	*D*—H⋯*A*
O1—H1⋯N1	0.82	1.98	2.7577 (18)	158
O2—H2⋯N2^i^	0.82	1.93	2.7279 (17)	164
O3—H3⋯N4^ii^	0.82	1.89	2.6816 (19)	162
O4—H4⋯N3	0.82	1.88	2.6525 (19)	156

Symmetry codes: (i) 
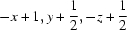
; (ii) 
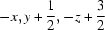
.

**Fig. 1.**
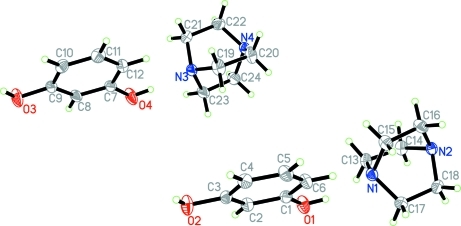
Asymmetric unit structure of the title compound. Displacement ellipsoids are drawn at the 30% probability level

**Fig. 2.**
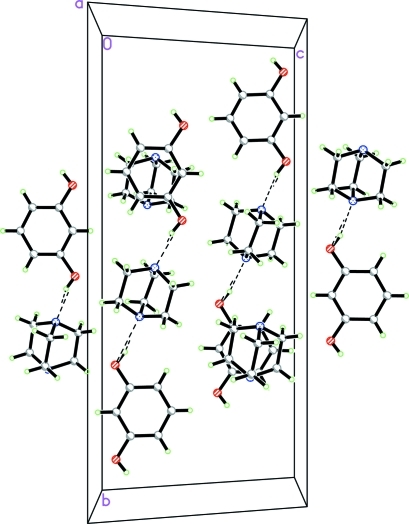
One-dimensional chain formation of the title compound constructed by hydrogen bonding (dashed lines).

**Fig. 3.**
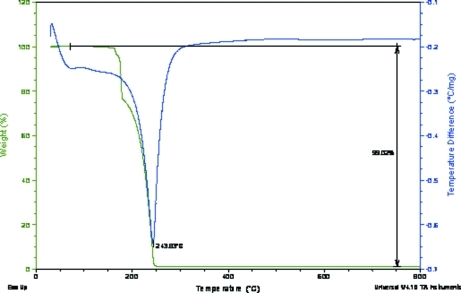
TG curve of the thermal decomposition of the title compound.

**Fig. 4.**
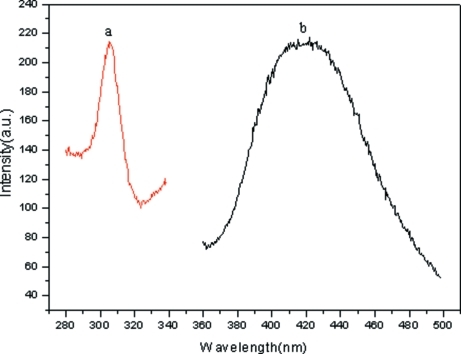
Solid state fluorescent excitation (a) and emission (b) of the title compound at room temperature.

## supplementary crystallographic information

### Comment

Alkali metal bis(salicylato)borates have received much attention, since lithium organoborates have been considered as the lithium battery electrolytes (Barthel *et al.*, 2000). In contrast, studies of organic base bis(salicylato)borates have been less extensive (Li and Liu, 2006; Han *et al.*, 2007). In the process of the synthesis of such compounds, a new crystal with supramolecular structure of [(C_6_H_6_O_2_)_2_ (C_6_H_12_N_2_)_2_] has been obtained.

Single crystal diffraction has revealed that the title compound crystallizes in the monoclinic space group P2_1_/c. It is composed of neutral resorcinol and triethylenediamine molecules (Fig.1), in which the resorcinol molecules came from the *in situ* decarboxylation of 2, 4-dihydroxy benzoic acid. These two kinds of molecules are connected by O—H···N hydrogen bonds to form one-dimensional supramolecular structure (Fig.2), with O···N distances in the range 2.6525 (19) – 2.7577 (18) Å and O—H···N angles in the range 156–164° (Table 1).

In order to evaluate the thermal stability of the synthesized compounds, TG experiments were employed under N_2_ atmosphere (Fig. 3). The crystal is stable before 150 °C, and quickly completes the decomposition process from 150 °C to 250 °C. The total weight loss of 99.02% corresponds to the self-decomposition of all organic matter (calculated value of 100%). The luminescent properties of this compound in the solid state at room temperature were investigated. As shown in Fig. 4, upon excitation of the solid sample at 300 nm, it exhibited strong fluorescent emission bands at 420 nm.

### Experimental

All reagents used in the synthesis were analytic grade and were used without further purification. A solution of boric acid (0.1684 g) in 2.5 ml distilled water was added to a stirred solution of 2, 4-dihydroxybenzoic acid (0.7706 g) in 10 ml of a mixed ethanol/water (1:1) solvent. The reaction mixture was stirred at 80 °C for 20 minutes, then 0.5507 g of triethylenediamine hexahydrate was added slowly. After 4 h continued heating and stirring, the obtained clear solution was then allowed to stand in an open beaker for several days at room temperature. The resulting colorless crystals were collected and dried in air at ambient temperature. IR (KBr pellets, cm^-1^): 458, 541, 686, 778, 837, 956, 1058, 1147, 1347, 1450, 1609, 1789, 2354, 2875, 2945 and 3054.

### Refinement

H atoms were placed in calculated positions and refined in riding mode with O—H = 0.820 Å and *U*_iso_(H) = 1.5*U*_eq_(O), C—H = 0.930–0.970 Å and *U*_iso_(H) = 1.2*U*_eq_(C).

### Crystal data

**Table:**

C_6_H_12_N_2_·C_6_H_6_O_2_	*F*(000) = 960
*M**_r_* = 222.29	*D*_x_ = 1.269 Mg m^−^^3^
Monoclinic, *P*2_1_/*c*	Mo *K*α radiation, λ = 0.71073 Å
*a* = 9.4882 (5) Å	Cell parameters from 4982 reflections
*b* = 23.7390 (11) Å	θ = 2.5–28.7°
*c* = 11.2532 (6) Å	µ = 0.09 mm^−^^1^
β = 113.335 (6)°	*T* = 293 K
*V* = 2327.3 (2) Å^3^	Block, colorless
*Z* = 8	0.45 × 0.43 × 0.02 mm

### Data collection

**Table:**

Oxford Diffraction Xcalibur Eos Gemini diffractometer	4091 independent reflections
Radiation source: fine-focus sealed tube	3270 reflections with *I* > 2σ(*I*)
graphite	*R*_int_ = 0.017
Detector resolution: 16.0356 pixels mm^-1^	θ_max_ = 25.0°, θ_min_ = 2.5°
φ and ω scans	*h* = −11→7
Absorption correction: multi-scan (*CrysAlis PRO*; Oxford Diffraction, 2010)	*k* = −25→28
*T*_min_ = 0.789, *T*_max_ = 1.000	*l* = −13→13
8717 measured reflections	

### Refinement

**Table:**

Refinement on *F*^2^	Secondary atom site location: difference Fourier map
Least-squares matrix: full	Hydrogen site location: inferred from neighbouring sites
*R*[*F*^2^ > 2σ(*F*^2^)] = 0.045	H-atom parameters constrained
*wR*(*F*^2^) = 0.121	*w* = 1/[σ^2^(*F*_o_^2^) + (0.0581*P*)^2^ + 0.6593*P*] where *P* = (*F*_o_^2^ + 2*F*_c_^2^)/3
*S* = 1.03	(Δ/σ)_max_ < 0.001
4091 reflections	Δρ_max_ = 0.19 e Å^−^^3^
290 parameters	Δρ_min_ = −0.16 e Å^−^^3^
0 restraints	Extinction correction: *SHELXL97* (Sheldrick, 2008), Fc^*^=kFc[1+0.001xFc^2^λ^3^/sin(2θ)]^-1/4^
Primary atom site location: structure-invariant direct methods	Extinction coefficient: 0.0037 (5)

### Special details

**Table:**

Geometry. All e.s.d.'s (except the e.s.d. in the dihedral angle between two l.s. planes) are estimated using the full covariance matrix. The cell e.s.d.'s are taken into account individually in the estimation of e.s.d.'s in distances, angles and torsion angles; correlations between e.s.d.'s in cell parameters are only used when they are defined by crystal symmetry. An approximate (isotropic) treatment of cell e.s.d.'s is used for estimating e.s.d.'s involving l.s. planes.
Refinement. Refinement of *F*^2^ against ALL reflections. The weighted *R*-factor *wR* and goodness of fit *S* are based on *F*^2^, conventional *R*-factors *R* are based on *F*, with *F* set to zero for negative *F*^2^. The threshold expression of *F*^2^ > σ(*F*^2^) is used only for calculating *R*-factors(gt) *etc*. and is not relevant to the choice of reflections for refinement. *R*-factors based on *F*^2^ are statistically about twice as large as those based on *F*, and *R*- factors based on ALL data will be even larger.

### Fractional atomic coordinates and isotropic or equivalent isotropic displacement parameters (Å^2^)

**Table:**

	*x*	*y*	*z*	*U*_iso_*/*U*_eq_	
N1	0.57689 (16)	0.11324 (6)	0.29731 (13)	0.0351 (3)	
N2	0.50838 (16)	0.01065 (5)	0.22286 (13)	0.0339 (3)	
N3	0.08210 (16)	0.23305 (6)	0.76867 (13)	0.0370 (3)	
N4	0.01256 (16)	0.12882 (6)	0.72564 (13)	0.0382 (4)	
O1	0.66383 (16)	0.21624 (5)	0.41752 (11)	0.0498 (4)	
H1	0.6499	0.1888	0.3698	0.075*	
O2	0.57485 (17)	0.41139 (5)	0.40869 (12)	0.0513 (4)	
H2	0.5375	0.4376	0.3585	0.077*	
O3	0.10750 (18)	0.52913 (5)	0.87710 (13)	0.0639 (5)	
H3	0.0696	0.5564	0.8308	0.096*	
O4	0.16822 (17)	0.33461 (5)	0.86965 (13)	0.0623 (4)	
H4	0.1466	0.3072	0.8214	0.093*	
C1	0.60465 (18)	0.26301 (7)	0.34498 (15)	0.0329 (4)	
C2	0.61811 (19)	0.31376 (7)	0.40953 (15)	0.0351 (4)	
H2A	0.6659	0.3149	0.4994	0.042*	
C3	0.56053 (19)	0.36304 (7)	0.34055 (15)	0.0334 (4)	
C4	0.4897 (2)	0.36123 (7)	0.20618 (16)	0.0383 (4)	
H4A	0.4502	0.3939	0.1593	0.046*	
C5	0.4784 (2)	0.31060 (7)	0.14309 (16)	0.0429 (4)	
H5	0.4320	0.3095	0.0532	0.051*	
C6	0.5348 (2)	0.26146 (7)	0.21091 (16)	0.0406 (4)	
H6	0.5261	0.2276	0.1670	0.049*	
C7	0.10691 (19)	0.38158 (7)	0.80009 (17)	0.0381 (4)	
C8	0.13280 (19)	0.43163 (7)	0.86713 (17)	0.0389 (4)	
H8	0.1890	0.4317	0.9562	0.047*	
C9	0.07617 (19)	0.48179 (7)	0.80370 (17)	0.0378 (4)	
C10	−0.0087 (2)	0.48160 (7)	0.67068 (17)	0.0412 (4)	
H10	−0.0467	0.5151	0.6268	0.049*	
C11	−0.0358 (2)	0.43120 (8)	0.60472 (17)	0.0449 (5)	
H11	−0.0936	0.4310	0.5159	0.054*	
C12	0.0208 (2)	0.38112 (8)	0.66717 (17)	0.0429 (4)	
H12	0.0017	0.3475	0.6212	0.051*	
C13	0.5384 (2)	0.08133 (7)	0.39281 (16)	0.0415 (4)	
H13A	0.4484	0.0977	0.4000	0.050*	
H13B	0.6229	0.0837	0.4769	0.050*	
C14	0.5066 (2)	0.01928 (7)	0.35205 (17)	0.0422 (4)	
H14A	0.5841	−0.0043	0.4147	0.051*	
H14B	0.4073	0.0084	0.3504	0.051*	
C15	0.4430 (2)	0.11111 (7)	0.17216 (17)	0.0445 (5)	
H15A	0.4681	0.1296	0.1061	0.053*	
H15B	0.3575	0.1311	0.1794	0.053*	
C16	0.3966 (2)	0.04980 (7)	0.13256 (17)	0.0444 (5)	
H16A	0.2957	0.0427	0.1321	0.053*	
H16B	0.3913	0.0435	0.0457	0.053*	
C17	0.7064 (2)	0.08497 (8)	0.2814 (2)	0.0471 (5)	
H17A	0.7935	0.0835	0.3642	0.056*	
H17B	0.7365	0.1063	0.2218	0.056*	
C18	0.6620 (2)	0.02515 (8)	0.2297 (2)	0.0458 (5)	
H18A	0.6632	0.0223	0.1441	0.055*	
H18B	0.7364	−0.0013	0.2859	0.055*	
C19	0.0785 (3)	0.21639 (8)	0.64220 (19)	0.0555 (5)	
H19A	0.1771	0.2244	0.6387	0.067*	
H19B	0.0009	0.2381	0.5748	0.067*	
C20	0.0428 (3)	0.15370 (8)	0.61859 (19)	0.0543 (5)	
H20A	−0.0462	0.1486	0.5382	0.065*	
H20B	0.1291	0.1346	0.6106	0.065*	
C21	−0.0700 (2)	0.22206 (8)	0.7684 (2)	0.0494 (5)	
H21A	−0.1456	0.2454	0.7035	0.059*	
H21B	−0.0699	0.2319	0.8521	0.059*	
C22	−0.1134 (2)	0.16008 (8)	0.7396 (2)	0.0514 (5)	
H22A	−0.1356	0.1440	0.8095	0.062*	
H22B	−0.2050	0.1570	0.6605	0.062*	
C23	0.1955 (2)	0.19796 (8)	0.86864 (19)	0.0491 (5)	
H23A	0.2009	0.2091	0.9533	0.059*	
H23B	0.2960	0.2036	0.8670	0.059*	
C24	0.1510 (2)	0.13541 (8)	0.84538 (19)	0.0491 (5)	
H24A	0.2349	0.1142	0.8386	0.059*	
H24B	0.1319	0.1207	0.9180	0.059*	

### Atomic displacement parameters (Å^2^)

**Table:**

	*U*^11^	*U*^22^	*U*^33^	*U*^12^	*U*^13^	*U*^23^
N1	0.0378 (8)	0.0284 (7)	0.0371 (7)	−0.0008 (6)	0.0126 (6)	−0.0023 (6)
N2	0.0394 (8)	0.0272 (7)	0.0350 (7)	−0.0009 (6)	0.0148 (6)	−0.0003 (6)
N3	0.0382 (8)	0.0283 (7)	0.0412 (8)	0.0004 (6)	0.0124 (6)	−0.0014 (6)
N4	0.0408 (8)	0.0281 (7)	0.0431 (8)	−0.0031 (6)	0.0138 (6)	−0.0005 (6)
O1	0.0681 (9)	0.0267 (6)	0.0416 (7)	0.0065 (6)	0.0080 (6)	0.0021 (5)
O2	0.0786 (10)	0.0290 (7)	0.0418 (7)	0.0095 (6)	0.0192 (7)	−0.0008 (5)
O3	0.0823 (11)	0.0259 (7)	0.0576 (9)	0.0054 (7)	0.0000 (7)	−0.0026 (6)
O4	0.0767 (10)	0.0246 (7)	0.0555 (8)	0.0021 (6)	−0.0060 (7)	0.0006 (6)
C1	0.0328 (9)	0.0274 (8)	0.0368 (9)	0.0013 (7)	0.0121 (7)	0.0033 (7)
C2	0.0390 (9)	0.0338 (9)	0.0303 (8)	0.0019 (7)	0.0113 (7)	0.0023 (7)
C3	0.0353 (9)	0.0282 (8)	0.0385 (9)	0.0001 (7)	0.0164 (7)	−0.0002 (7)
C4	0.0398 (10)	0.0328 (9)	0.0388 (9)	0.0035 (8)	0.0118 (7)	0.0084 (7)
C5	0.0495 (11)	0.0419 (10)	0.0300 (9)	−0.0025 (8)	0.0081 (7)	0.0003 (7)
C6	0.0460 (10)	0.0330 (9)	0.0378 (9)	−0.0034 (8)	0.0115 (8)	−0.0049 (7)
C7	0.0325 (9)	0.0282 (9)	0.0466 (10)	−0.0003 (7)	0.0082 (7)	0.0034 (7)
C8	0.0371 (9)	0.0299 (9)	0.0394 (9)	−0.0019 (7)	0.0043 (7)	0.0022 (7)
C9	0.0343 (9)	0.0275 (9)	0.0474 (10)	−0.0009 (7)	0.0119 (7)	0.0007 (7)
C10	0.0426 (10)	0.0352 (10)	0.0456 (10)	0.0087 (8)	0.0171 (8)	0.0102 (8)
C11	0.0466 (11)	0.0503 (11)	0.0350 (9)	0.0052 (9)	0.0131 (8)	0.0034 (8)
C12	0.0445 (10)	0.0359 (10)	0.0460 (10)	−0.0003 (8)	0.0156 (8)	−0.0063 (8)
C13	0.0528 (11)	0.0383 (10)	0.0364 (9)	0.0007 (8)	0.0208 (8)	−0.0038 (7)
C14	0.0551 (11)	0.0350 (10)	0.0411 (10)	−0.0009 (8)	0.0241 (8)	0.0031 (7)
C15	0.0492 (11)	0.0317 (9)	0.0422 (10)	0.0027 (8)	0.0071 (8)	0.0033 (8)
C16	0.0444 (10)	0.0371 (10)	0.0388 (10)	−0.0023 (8)	0.0029 (8)	0.0010 (8)
C17	0.0399 (10)	0.0425 (11)	0.0629 (12)	−0.0079 (8)	0.0246 (9)	−0.0122 (9)
C18	0.0452 (11)	0.0411 (11)	0.0574 (11)	0.0012 (9)	0.0271 (9)	−0.0091 (9)
C19	0.0784 (15)	0.0443 (11)	0.0535 (12)	−0.0100 (11)	0.0365 (11)	0.0010 (9)
C20	0.0795 (15)	0.0443 (11)	0.0462 (11)	−0.0066 (10)	0.0326 (10)	−0.0112 (9)
C21	0.0422 (10)	0.0457 (11)	0.0599 (12)	0.0057 (9)	0.0198 (9)	−0.0080 (9)
C22	0.0396 (10)	0.0475 (11)	0.0700 (13)	−0.0047 (9)	0.0247 (10)	−0.0018 (10)
C23	0.0402 (10)	0.0382 (10)	0.0533 (11)	0.0008 (8)	0.0020 (8)	−0.0026 (8)
C24	0.0467 (11)	0.0348 (10)	0.0535 (11)	0.0047 (8)	0.0067 (9)	0.0050 (8)

### Geometric parameters (Å, °)

**Table:**

N1—C17	1.472 (2)	C10—C11	1.377 (3)
N1—C13	1.474 (2)	C10—H10	0.9300
N1—C15	1.478 (2)	C11—C12	1.377 (2)
N2—C18	1.469 (2)	C11—H11	0.9300
N2—C16	1.472 (2)	C12—H12	0.9300
N2—C14	1.475 (2)	C13—C14	1.537 (2)
N3—C21	1.465 (2)	C13—H13A	0.9700
N3—C19	1.464 (2)	C13—H13B	0.9700
N3—C23	1.469 (2)	C14—H14A	0.9700
N4—C24	1.471 (2)	C14—H14B	0.9700
N4—C22	1.467 (2)	C15—C16	1.535 (2)
N4—C20	1.468 (2)	C15—H15A	0.9700
O1—C1	1.3605 (19)	C15—H15B	0.9700
O1—H1	0.8200	C16—H16A	0.9700
O2—C3	1.3569 (19)	C16—H16B	0.9700
O2—H2	0.8200	C17—C18	1.529 (2)
O3—C9	1.356 (2)	C17—H17A	0.9700
O3—H3	0.8200	C17—H17B	0.9700
O4—C7	1.354 (2)	C18—H18A	0.9700
O4—H4	0.8200	C18—H18B	0.9700
C1—C6	1.387 (2)	C19—C20	1.526 (3)
C1—C2	1.386 (2)	C19—H19A	0.9700
C2—C3	1.391 (2)	C19—H19B	0.9700
C2—H2A	0.9300	C20—H20A	0.9700
C3—C4	1.391 (2)	C20—H20B	0.9700
C4—C5	1.378 (2)	C21—C22	1.528 (3)
C4—H4A	0.9300	C21—H21A	0.9700
C5—C6	1.381 (2)	C21—H21B	0.9700
C5—H5	0.9300	C22—H22A	0.9700
C6—H6	0.9300	C22—H22B	0.9700
C7—C8	1.376 (2)	C23—C24	1.538 (2)
C7—C12	1.392 (2)	C23—H23A	0.9700
C8—C9	1.383 (2)	C23—H23B	0.9700
C8—H8	0.9300	C24—H24A	0.9700
C9—C10	1.391 (2)	C24—H24B	0.9700
			
C17—N1—C13	108.30 (14)	C13—C14—H14B	109.6
C17—N1—C15	108.24 (14)	H14A—C14—H14B	108.1
C13—N1—C15	108.02 (14)	N1—C15—C16	110.39 (13)
C18—N2—C16	108.44 (14)	N1—C15—H15A	109.6
C18—N2—C14	108.32 (14)	C16—C15—H15A	109.6
C16—N2—C14	107.85 (14)	N1—C15—H15B	109.6
C21—N3—C19	107.84 (15)	C16—C15—H15B	109.6
C21—N3—C23	108.82 (15)	H15A—C15—H15B	108.1
C19—N3—C23	108.61 (15)	N2—C16—C15	110.69 (13)
C24—N4—C22	108.51 (15)	N2—C16—H16A	109.5
C24—N4—C20	108.35 (15)	C15—C16—H16A	109.5
C22—N4—C20	108.48 (15)	N2—C16—H16B	109.5
C1—O1—H1	109.5	C15—C16—H16B	109.5
C3—O2—H2	109.5	H16A—C16—H16B	108.1
C9—O3—H3	109.5	N1—C17—C18	110.62 (14)
C7—O4—H4	109.5	N1—C17—H17A	109.5
O1—C1—C6	122.46 (15)	C18—C17—H17A	109.5
O1—C1—C2	117.66 (14)	N1—C17—H17B	109.5
C6—C1—C2	119.87 (15)	C18—C17—H17B	109.5
C1—C2—C3	120.29 (14)	H17A—C17—H17B	108.1
C1—C2—H2A	119.9	N2—C18—C17	110.82 (14)
C3—C2—H2A	119.9	N2—C18—H18A	109.5
O2—C3—C2	117.83 (14)	C17—C18—H18A	109.5
O2—C3—C4	122.52 (15)	N2—C18—H18B	109.5
C2—C3—C4	119.64 (15)	C17—C18—H18B	109.5
C5—C4—C3	119.50 (15)	H18A—C18—H18B	108.1
C5—C4—H4A	120.2	N3—C19—C20	110.52 (15)
C3—C4—H4A	120.2	N3—C19—H19A	109.5
C6—C5—C4	121.20 (15)	C20—C19—H19A	109.5
C6—C5—H5	119.4	N3—C19—H19B	109.5
C4—C5—H5	119.4	C20—C19—H19B	109.5
C5—C6—C1	119.50 (16)	H19A—C19—H19B	108.1
C5—C6—H6	120.3	N4—C20—C19	110.57 (14)
C1—C6—H6	120.3	N4—C20—H20A	109.5
O4—C7—C8	116.83 (15)	C19—C20—H20A	109.5
O4—C7—C12	123.35 (15)	N4—C20—H20B	109.5
C8—C7—C12	119.82 (15)	C19—C20—H20B	109.5
C7—C8—C9	120.79 (15)	H20A—C20—H20B	108.1
C7—C8—H8	119.6	N3—C21—C22	110.75 (15)
C9—C8—H8	119.6	N3—C21—H21A	109.5
O3—C9—C8	116.91 (15)	C22—C21—H21A	109.5
O3—C9—C10	123.51 (15)	N3—C21—H21B	109.5
C8—C9—C10	119.58 (16)	C22—C21—H21B	109.5
C11—C10—C9	119.20 (16)	H21A—C21—H21B	108.1
C11—C10—H10	120.4	N4—C22—C21	110.34 (15)
C9—C10—H10	120.4	N4—C22—H22A	109.6
C10—C11—C12	121.54 (16)	C21—C22—H22A	109.6
C10—C11—H11	119.2	N4—C22—H22B	109.6
C12—C11—H11	119.2	C21—C22—H22B	109.6
C11—C12—C7	119.07 (16)	H22A—C22—H22B	108.1
C11—C12—H12	120.5	N3—C23—C24	110.40 (14)
C7—C12—H12	120.5	N3—C23—H23A	109.6
N1—C13—C14	110.55 (13)	C24—C23—H23A	109.6
N1—C13—H13A	109.5	N3—C23—H23B	109.6
C14—C13—H13A	109.5	C24—C23—H23B	109.6
N1—C13—H13B	109.5	H23A—C23—H23B	108.1
C14—C13—H13B	109.5	N4—C24—C23	110.13 (14)
H13A—C13—H13B	108.1	N4—C24—H24A	109.6
N2—C14—C13	110.48 (14)	C23—C24—H24A	109.6
N2—C14—H14A	109.6	N4—C24—H24B	109.6
C13—C14—H14A	109.6	C23—C24—H24B	109.6
N2—C14—H14B	109.6	H24A—C24—H24B	108.1

### Hydrogen-bond geometry (Å, °)

**Table:**

*D*—H···*A*	*D*—H	H···*A*	*D*···*A*	*D*—H···*A*
O1—H1···N1	0.82	1.98	2.7577 (18)	158
O2—H2···N2^i^	0.82	1.93	2.7279 (17)	164
O3—H3···N4^ii^	0.82	1.89	2.6816 (19)	162
O4—H4···N3	0.82	1.88	2.6525 (19)	156

Symmetry codes: (i) −*x*+1, *y*+1/2, −*z*+1/2; (ii) −*x*, *y*+1/2, −*z*+3/2.

## References

[bb1] Barthel, J., Schmid, A. & Gores, H. J. (2000). *J. Electrochem. Soc.* **147**, 21–24.

[bb2] Han, W.-H., Li, P. & Liu, Z.-H. (2007). *Acta Cryst.* E**63**, o3946.

[bb3] Li, P. & Liu, Z.-H. (2006). *Z. Kristallogr. New Cryst. Struct.* **221**, 179–180.

[bb4] Oxford Diffraction (2010). *CrysAlis PRO* Oxford Diffraction Ltd, Yarnton, England.

[bb5] Sheldrick, G. M. (2008). *Acta Cryst.* A**64**, 112–122. 10.1107/S0108767307043930 18156677

[bb6] Westrip, S. P. (2010). *J. Appl. Cryst.* **43**, 920–925.

